# *N*,*N*′-Bis(2-hydr­oxy-3-eth­oxybenzyl­idene)butane-1,4-diamine

**DOI:** 10.1107/S1600536809007545

**Published:** 2009-03-06

**Authors:** Hoong-Kun Fun, Reza Kia, Hadi Kargar, Arezoo Jamshidvand

**Affiliations:** a X-ray Crystallography Unit, School of Physics, Universiti Sains Malaysia, 11800 USM, Penang, Malaysia; b Department of Chemistry, School of Science, Payame Noor University (PNU), Ardakan, Yazd, Iran

## Abstract

The title Schiff base compound, C_22_H_28_N_2_O_4_, lies across a crystallographic inversion centre and adopts an *E* configuration with respect to the C=N bond. Pairs of weak inter­molecular C—H⋯O inter­actions link neighbouring mol­ecules into dimers with an *R* ^2^_2_(28) ring motif. The crystal structure is stabilized by inter­molecular C—H⋯π inter­actions. An intramolecular O—H⋯N hydrogen bond occurs.

## Related literature

For hydrogen-bond motifs, see: Bernstein *et al.* (1995 ▶). For information on Schiff base ligands and complexes and their applications, see, for example: Calligaris & Randaccio (1987 ▶); Casellato & Vigato (1977 ▶); Fun & Kia (2008**a* ▶,b* ▶). For the stability of the temperature controller used for the data collection, see: Cosier & Glazer (1986 ▶).
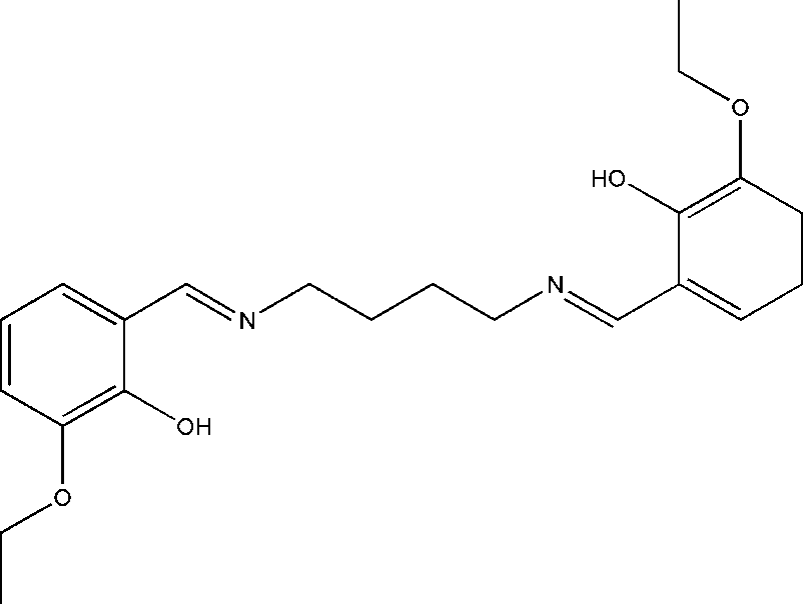


## Experimental

### 

#### Crystal data

C_22_H_28_N_2_O_4_
*M* *_r_* = 384.46
Triclinic, 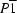

*a* = 6.8647 (2) Å
*b* = 6.9052 (2) Å
*c* = 10.8083 (3) Å
α = 92.779 (2)°
β = 99.908 (2)°
γ = 101.239 (2)°
*V* = 493.23 (2) Å^3^
*Z* = 1
Mo *K*α radiation
μ = 0.09 mm^−1^
*T* = 100 K
0.45 × 0.19 × 0.07 mm

#### Data collection

Bruker SMART APEXII CCD area-detector diffractometer
Absorption correction: multi-scan (*SADABS*; Bruker, 2005 ▶) *T*_min_ = 0.961, *T*_max_ = 0.994
8962 measured reflections
2954 independent reflections
2157 reflections with *I* > 2σ(*I*)
*R*_int_ = 0.033

#### Refinement

*R*[*F* ^2^ > 2σ(*F* ^2^)] = 0.049
*wR*(*F* ^2^) = 0.131
*S* = 1.04
2954 reflections
129 parameters
H-atom parameters constrained
Δρ_max_ = 0.43 e Å^−3^
Δρ_min_ = −0.27 e Å^−3^

### 

Data collection: *APEX2* (Bruker, 2005 ▶); cell refinement: *SAINT* (Bruker, 2005 ▶); data reduction: *SAINT*; program(s) used to solve structure: *SHELXTL* (Sheldrick, 2008 ▶); program(s) used to refine structure: *SHELXTL*; molecular graphics: *SHELXTL*; software used to prepare material for publication: *SHELXTL* and *PLATON* (Spek, 2009 ▶).

## Supplementary Material

Crystal structure: contains datablocks global, I. DOI: 10.1107/S1600536809007545/at2734sup1.cif

Structure factors: contains datablocks I. DOI: 10.1107/S1600536809007545/at2734Isup2.hkl

Additional supplementary materials:  crystallographic information; 3D view; checkCIF report

## Figures and Tables

**Table 1:** Hydrogen-bond geometry (Å, °)

*D*—H⋯*A*	*D*—H	H⋯*A*	*D*⋯*A*	*D*—H⋯*A*
O1—H1⋯N1	0.84	1.82	2.5638 (14)	147
C5—H5*A*⋯O1^i^	0.95	2.59	3.2268 (14)	125
C11—H11*B*⋯*Cg*1^ii^	0.98	2.96	3.5403 (15)	145

Symmetry codes: (i) 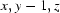; (ii) 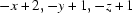. *Cg*1 is the centroid of the C1–C6 benzene ring.

**Fig. 1.**
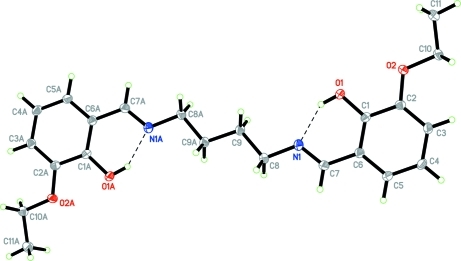
The molecular structure of the title compound with atom labels and 50% probability ellipsoids for non-H atoms. The suffix A corresponds to symmetry code (-x + 2, -y + 1, -z + 2).

**Fig. 2.**
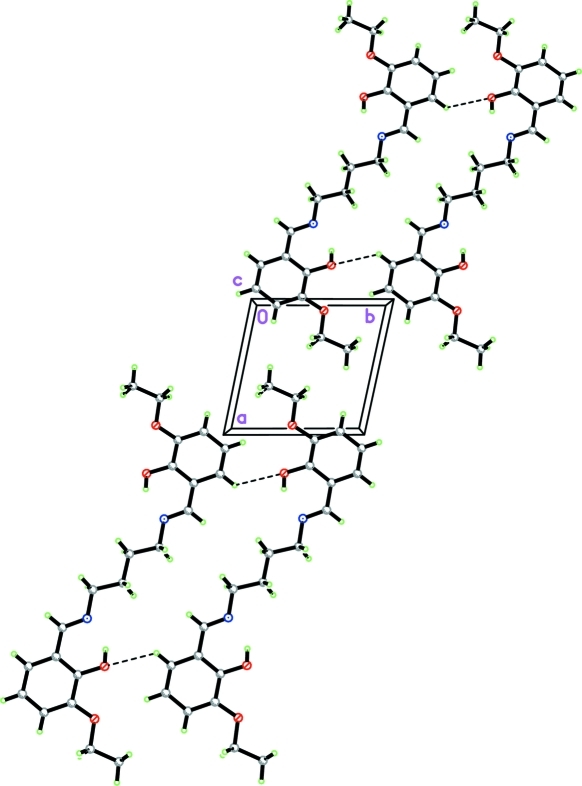
The crystal packing of the title compound, viewed down the c axis showing dimer formation by R22(28) ring motif.

## supplementary crystallographic information

### Comment

The condensation of primary amines with carbonyl compounds yields Schiff base (Casellato & Vigato, 1977) that are still one of the most prevalent mixed-donor ligands in coordination chemistry. In the past two decades, the synthesis, structure and properties of Schiff base complexes have stimulated much interest for their noteworthy contributions in single molecule-based magnetism, materials science, catalysis of many reactions like carbonylation, hydroformylation, reduction, oxidation, epoxidation and hydrolysis (Casellato & Vigato 1977). In comparison to the Schiff base metal complexes, only a relatively small number of free Schiff base ligands have been characterized (Calligaris & Randaccio, 1987). As an extension of our work (Fun & Kia 2008*a,b*) on the structural characterization of Schiff base ligands, the title compound is reported here.

The molecule of the title compound, Fig 1, lies across a crystallographic inversion centre and adopts an *E* configuration with respect to the azomethine (C═N) bond. The asymmetric unit of the compound is composed of one-half of the molecule. The imino group is coplanar with the benzene ring. Pairs of intermolecular C—H···O interactions link neighbouring molecules into dimers with a R^2^_2_(28) ring motif (Bernstein *et al.*, 1995). The crystal structure is stabilized by intermolecular C—H···π interactions [*Cg*1 is the centroid of the C1–C6 benzene ring] (Table 1).

### Experimental

The synthetic method has been described earlier (Fun, Kia & Kargar *et al.*, 2008*b*), except that 2-hydroxy-3-ethoxysalicylaldehyde was used . Single crystals suitable for *X*-ray diffraction were obtained by evaporation of an ethanol solution at room temperature.

### Refinement

H atom of the hydroxy group was positioned by a freely rotating O—H bond and constrained with a fixed distance of 0.84 Å. The rest of the hydrogen atoms were positioned geometrically with a riding model approximation with C—H = 0.95-0.99 Å and U_iso_(H) = 1.2 or 1.5 (C & O). A rotating group model was used for methyl group.

### Crystal data

**Table:**

C_22_H_28_N_2_O_4_	*Z* = 1
*M**_r_* = 384.46	*F*(000) = 206
Triclinic, *P*1	*D*_x_ = 1.294 Mg m^−^^3^
Hall symbol: -P 1	Mo *K*α radiation, λ = 0.71073 Å
*a* = 6.8647 (2) Å	Cell parameters from 2475 reflections
*b* = 6.9052 (2) Å	θ = 2.5–29.4°
*c* = 10.8083 (3) Å	µ = 0.09 mm^−^^1^
α = 92.779 (2)°	*T* = 100 K
β = 99.908 (2)°	Plate, yellow
γ = 101.239 (2)°	0.45 × 0.19 × 0.07 mm
*V* = 493.23 (2) Å^3^	

### Data collection

**Table:**

Bruker SMART APEXII CCD area-detector diffractometer	2954 independent reflections
Radiation source: fine-focus sealed tube	2157 reflections with *I* > 2σ(*I*)
graphite	*R*_int_ = 0.033
φ and ω scans	θ_max_ = 30.4°, θ_min_ = 1.9°
Absorption correction: multi-scan (*SADABS*; Bruker, 2005)	*h* = −9→9
*T*_min_ = 0.961, *T*_max_ = 0.994	*k* = −9→9
8962 measured reflections	*l* = −14→15

### Refinement

**Table:**

Refinement on *F*^2^	Primary atom site location: structure-invariant direct methods
Least-squares matrix: full	Secondary atom site location: difference Fourier map
*R*[*F*^2^ > 2σ(*F*^2^)] = 0.049	Hydrogen site location: inferred from neighbouring sites
*wR*(*F*^2^) = 0.131	H-atom parameters constrained
*S* = 1.04	*w* = 1/[σ^2^(*F*_o_^2^) + (0.0681*P*)^2^ + 0.047*P*] where *P* = (*F*_o_^2^ + 2*F*_c_^2^)/3
2954 reflections	(Δ/σ)_max_ < 0.001
129 parameters	Δρ_max_ = 0.43 e Å^−^^3^
0 restraints	Δρ_min_ = −0.27 e Å^−^^3^

### Special details

**Table:**

Experimental. The crystal was placed in the cold stream of an Oxford Cyrosystems Cobra open-flow nitrogen cryostat (Cosier & Glazer, 1986) operating at 100.0 (1)K.
Geometry. All esds (except the esd in the dihedral angle between two l.s. planes) are estimated using the full covariance matrix. The cell esds are taken into account individually in the estimation of esds in distances, angles and torsion angles; correlations between esds in cell parameters are only used when they are defined by crystal symmetry. An approximate (isotropic) treatment of cell esds is used for estimating esds involving l.s. planes.
Refinement. Refinement of F^2^ against ALL reflections. The weighted R-factor wR and goodness of fit S are based on F^2^, conventional R-factors R are based on F, with F set to zero for negative F^2^. The threshold expression of F^2^ > 2sigma(F^2^) is used only for calculating R-factors(gt) etc. and is not relevant to the choice of reflections for refinement. R-factors based on F^2^ are statistically about twice as large as those based on F, and R- factors based on ALL data will be even larger.

### Fractional atomic coordinates and isotropic or equivalent isotropic displacement parameters (Å^2^)

**Table:**

	*x*	*y*	*z*	*U*_iso_*/*U*_eq_	
O1	0.69496 (13)	0.51130 (12)	0.23213 (9)	0.0191 (2)	
H1	0.5792	0.4902	0.1870	0.029*	
O2	1.05734 (12)	0.52787 (12)	0.36073 (8)	0.0178 (2)	
N1	0.35928 (15)	0.30726 (15)	0.10848 (10)	0.0181 (2)	
C1	0.75200 (17)	0.33702 (16)	0.25124 (11)	0.0149 (2)	
C2	0.94642 (17)	0.34181 (16)	0.32117 (11)	0.0157 (2)	
C3	1.00920 (18)	0.16571 (17)	0.34438 (12)	0.0177 (2)	
H3A	1.1398	0.1690	0.3921	0.021*	
C4	0.88179 (18)	−0.01729 (17)	0.29812 (12)	0.0188 (3)	
H4A	0.9259	−0.1372	0.3146	0.023*	
C5	0.69162 (18)	−0.02237 (17)	0.22845 (12)	0.0178 (2)	
H5A	0.6058	−0.1462	0.1964	0.021*	
C6	0.62455 (17)	0.15406 (16)	0.20476 (11)	0.0157 (2)	
C7	0.42169 (18)	0.14831 (17)	0.13323 (11)	0.0169 (2)	
H7A	0.3346	0.0238	0.1046	0.020*	
C8	0.15522 (17)	0.29668 (17)	0.03663 (12)	0.0185 (3)	
H8A	0.0561	0.2041	0.0735	0.022*	
H8B	0.1478	0.2459	−0.0517	0.022*	
C9	0.10389 (17)	0.50109 (17)	0.03934 (11)	0.0170 (2)	
H9A	0.2076	0.5946	0.0065	0.020*	
H9B	0.1064	0.5488	0.1276	0.020*	
C10	1.26442 (17)	0.54481 (17)	0.42136 (12)	0.0182 (3)	
H10A	1.3370	0.4755	0.3676	0.022*	
H10B	1.2713	0.4857	0.5032	0.022*	
C11	1.35712 (19)	0.76329 (19)	0.44083 (13)	0.0227 (3)	
H11A	1.5003	0.7823	0.4795	0.034*	
H11B	1.2866	0.8291	0.4964	0.034*	
H11C	1.3448	0.8205	0.3593	0.034*	

### Atomic displacement parameters (Å^2^)

**Table:**

	*U*^11^	*U*^22^	*U*^33^	*U*^12^	*U*^13^	*U*^23^
O1	0.0164 (4)	0.0136 (4)	0.0265 (5)	0.0048 (3)	−0.0003 (3)	0.0029 (3)
O2	0.0134 (4)	0.0146 (4)	0.0234 (5)	0.0009 (3)	0.0006 (3)	0.0005 (3)
N1	0.0145 (5)	0.0188 (5)	0.0215 (5)	0.0055 (4)	0.0021 (4)	0.0024 (4)
C1	0.0163 (6)	0.0129 (5)	0.0168 (6)	0.0040 (4)	0.0054 (4)	0.0031 (4)
C2	0.0154 (6)	0.0149 (5)	0.0172 (6)	0.0021 (4)	0.0051 (4)	0.0015 (4)
C3	0.0143 (6)	0.0190 (6)	0.0203 (6)	0.0050 (4)	0.0023 (5)	0.0029 (4)
C4	0.0194 (6)	0.0149 (5)	0.0237 (6)	0.0069 (4)	0.0044 (5)	0.0039 (4)
C5	0.0175 (6)	0.0133 (5)	0.0223 (6)	0.0030 (4)	0.0029 (5)	0.0015 (4)
C6	0.0142 (6)	0.0157 (5)	0.0176 (6)	0.0038 (4)	0.0033 (4)	0.0021 (4)
C7	0.0154 (6)	0.0154 (5)	0.0192 (6)	0.0019 (4)	0.0027 (5)	0.0011 (4)
C8	0.0137 (6)	0.0187 (6)	0.0221 (6)	0.0043 (4)	0.0001 (5)	0.0009 (5)
C9	0.0157 (6)	0.0176 (5)	0.0186 (6)	0.0055 (4)	0.0032 (5)	0.0025 (4)
C10	0.0128 (6)	0.0193 (6)	0.0221 (6)	0.0029 (4)	0.0030 (5)	0.0009 (4)
C11	0.0150 (6)	0.0219 (6)	0.0291 (7)	0.0006 (4)	0.0021 (5)	0.0027 (5)

### Geometric parameters (Å, °)

**Table:**

O1—C1	1.3507 (12)	C6—C7	1.4641 (15)
O1—H1	0.8400	C7—H7A	0.9500
O2—C2	1.3662 (13)	C8—C9	1.5201 (15)
O2—C10	1.4391 (13)	C8—H8A	0.9900
N1—C7	1.2776 (14)	C8—H8B	0.9900
N1—C8	1.4666 (14)	C9—C9^i^	1.527 (2)
C1—C6	1.4037 (16)	C9—H9A	0.9900
C1—C2	1.4096 (16)	C9—H9B	0.9900
C2—C3	1.3871 (15)	C10—C11	1.5081 (17)
C3—C4	1.4033 (17)	C10—H10A	0.9900
C3—H3A	0.9500	C10—H10B	0.9900
C4—C5	1.3833 (16)	C11—H11A	0.9800
C4—H4A	0.9500	C11—H11B	0.9800
C5—C6	1.4036 (15)	C11—H11C	0.9800
C5—H5A	0.9500		
			
C1—O1—H1	109.5	N1—C8—C9	109.97 (10)
C2—O2—C10	117.43 (9)	N1—C8—H8A	109.7
C7—N1—C8	120.11 (11)	C9—C8—H8A	109.7
O1—C1—C6	122.32 (10)	N1—C8—H8B	109.7
O1—C1—C2	118.03 (10)	C9—C8—H8B	109.7
C6—C1—C2	119.65 (10)	H8A—C8—H8B	108.2
O2—C2—C3	125.83 (11)	C8—C9—C9^i^	111.80 (13)
O2—C2—C1	114.48 (9)	C8—C9—H9A	109.3
C3—C2—C1	119.70 (11)	C9^i^—C9—H9A	109.3
C2—C3—C4	120.70 (11)	C8—C9—H9B	109.3
C2—C3—H3A	119.7	C9^i^—C9—H9B	109.3
C4—C3—H3A	119.7	H9A—C9—H9B	107.9
C5—C4—C3	119.72 (11)	O2—C10—C11	106.44 (9)
C5—C4—H4A	120.1	O2—C10—H10A	110.4
C3—C4—H4A	120.1	C11—C10—H10A	110.4
C4—C5—C6	120.48 (11)	O2—C10—H10B	110.4
C4—C5—H5A	119.8	C11—C10—H10B	110.4
C6—C5—H5A	119.8	H10A—C10—H10B	108.6
C5—C6—C1	119.75 (10)	C10—C11—H11A	109.5
C5—C6—C7	120.42 (11)	C10—C11—H11B	109.5
C1—C6—C7	119.83 (10)	H11A—C11—H11B	109.5
N1—C7—C6	121.38 (11)	C10—C11—H11C	109.5
N1—C7—H7A	119.3	H11A—C11—H11C	109.5
C6—C7—H7A	119.3	H11B—C11—H11C	109.5
			
C10—O2—C2—C3	6.39 (17)	C4—C5—C6—C7	−178.73 (11)
C10—O2—C2—C1	−173.76 (10)	O1—C1—C6—C5	−179.51 (11)
O1—C1—C2—O2	−0.84 (16)	C2—C1—C6—C5	0.09 (17)
C6—C1—C2—O2	179.55 (10)	O1—C1—C6—C7	−0.25 (17)
O1—C1—C2—C3	179.02 (10)	C2—C1—C6—C7	179.35 (10)
C6—C1—C2—C3	−0.60 (17)	C8—N1—C7—C6	−179.99 (10)
O2—C2—C3—C4	−179.65 (11)	C5—C6—C7—N1	−178.57 (11)
C1—C2—C3—C4	0.51 (18)	C1—C6—C7—N1	2.18 (18)
C2—C3—C4—C5	0.10 (18)	C7—N1—C8—C9	170.13 (11)
C3—C4—C5—C6	−0.61 (18)	N1—C8—C9—C9^i^	177.44 (12)
C4—C5—C6—C1	0.51 (18)	C2—O2—C10—C11	173.37 (10)

Symmetry codes: (i) −*x*, −*y*+1, −*z*.

### Hydrogen-bond geometry (Å, °)

**Table:**

*D*—H···*A*	*D*—H	H···*A*	*D*···*A*	*D*—H···*A*
O1—H1···N1	0.84	1.82	2.5638 (14)	147
C5—H5A···O1^ii^	0.95	2.59	3.2268 (14)	125
C11—H11B···Cg1^iii^	0.98	2.96	3.5403 (15)	145

Symmetry codes: (ii) *x*, *y*−1, *z*; (iii) −*x*+2, −*y*+1, −*z*+1.
